# Different Modulatory Effects of Cognitive Training and Aerobic Exercise on Resting State Functional Connectivity of Entorhinal Cortex in Community-Dwelling Older Adults

**DOI:** 10.3389/fnagi.2021.655245

**Published:** 2021-05-31

**Authors:** NanNan Gu, Hechun Li, Xinyi Cao, Ting Li, Lijuan Jiang, Han Zhang, Binglei Zhao, Cheng Luo, Chunbo Li

**Affiliations:** ^1^Shanghai Key Laboratory of Psychotic Disorders, Shanghai Mental Health Center, Shanghai Jiao Tong University School of Medicine, Shanghai, China; ^2^The Clinical Hospital of Chengdu Brain Science Institute, MOE Key Lab for Neuroinformation, Center for Information in Medicine, School of Life Sciences and Technology, University of Electronic Science and Technology of China, Chengdu, China; ^3^Clinical Neurocognitive Research Center, Shanghai Key Laboratory of Psychotic Disorders, Shanghai Mental Health Center, Shanghai Jiao Tong University School of Medicine, Shanghai, China; ^4^Department of Geriatric Psychiatry, Shanghai Changning Mental Health Center, Shanghai, China; ^5^Institute of Brain-Intelligence Technology, Zhangjiang Lab, Shanghai, China; ^6^Institute of Psychology and Behavioral Science, Shanghai Jiao Tong University, Shanghai, China; ^7^Center for Excellence in Brain Science and Intelligence Technology (CEBSIT), Chinese Academy of Sciences, Shanghai, China; ^8^Brain Science and Technology Research Center, Shanghai Jiao Tong University, Shanghai, China

**Keywords:** cognitive training, aerobic exercise, entorhinal cortex, resting state functional connectivity, older adults

## Abstract

The entorhinal cortex (EC) plays an essential role in age-related cognitive decline. However, the effect of functional connectivity (FC) changes between EC and other cerebral cortices on cognitive function remains unclear. The aim of this study was to explore the modulation of two interventions (cognitive training and aerobic exercise) on EC-FC in community-dwelling older adults. In total, 94 healthy older adults aged between 65 and 75 years were assigned to either the cognitive training or aerobic exercise group to receive 24 sessions over 12 weeks, or to a control group. Resting-state functional magnetic resonance imaging was performed at both baseline and 12-month follow-up. Compared to the cognitive training group, the aerobic exercise group showed greater EC-FC in the bilateral middle temporal gyrus, right supramarginal gyrus, left angular gyrus, and right postcentral gyrus. Compared to the control group, the cognitive training group had a decreased EC-FC in the right hippocampus, right middle temporal gyrus, left angular gyrus, and right postcentral gyrus and an increased EC-FC in the bilateral pallidum, while the aerobic exercise group showed increased EC-FC between the right medial prefrontal cortex(mPFC), bilateral pallidum, and right precuneus. Baseline EC-FC in the mPFC was positively correlated with the visuospatial/constructional index score of the Repeatable Battery for the Assessment of Neuropsychological Status. In the cognitive training group, EC-FC value changes in the right hippocampus were negatively correlated with changes in the RBANS delayed memory index score, while in the aerobic exercise group, EC-FC value changes in the left angular gyrus were positively correlated with changes in the RBANS attention index score. These findings support the hypothesis that both cognitive training and aerobic exercise can modulate EC-FC in aging populations but through different neural pathways.

## Introduction

Aging is accompanied with a decline in cognitive functions such as attention, processing speed, executive function, memory and reasoning (Drag and Bieliauskas, 2010). The aging of the global population has increased the prevalence of dementia, which is caused by diseases such as Alzheimer’s disease (AD), which imposes huge healthcare burdens on society and decreases the quality of life of older adults. Therefore, it is urgent to explore the pathophysiological mechanism of cognitive aging and identify effective interventions to delay the aging process. Although aging is accompanied with an alteration in the brain structure and function, increasing numbers of studies have shown that the aging brain maintains an impressive level of plasticity (Park and Reuter-Lorenz, 2009; Bherer, 2015).

Cognitive training is a promising approach to delay age-related cognitive decline (Kelly et al., 2014). Previous studies have demonstrated that cognitive training can improve memory, visual reasoning, visuospatial construction and attention in community-dwelling older adults (Mahncke et al., 2006; Cheng et al., 2012). The activity of memory-related regions, such as the frontal lobe, parietal lobe and bilateral hippocampus, is increased after cognitive training in healthy older adults and patients with mild cognitive impairment (MCI) (Belleville et al., 2011). Cognitive training increased functional connectivity (FC) between the right posterior cingulate cortex (PCC) and the left precuneus, superior parietal and temporal cortices (De Marco et al., 2016). Aerobic exercise is also reportedly beneficial for improving cognitive function, such as motor function, processing speed, and auditory and visual attention (Angevaren et al., 2008). Aerobic exercise primarily affects areas of the brain that are vulnerable to neurodegeneration, including the frontal, temporal and parietal lobes, the hippocampus/parahippocampal region, precuneus, anterior cingulate gyrus, and prefrontal cortex (PFC) (Haeger et al., 2019). Changes in body fitness, hippocampal perfusion, and hippocampal volume are positively related to changes in recognition memory and early recall for complex spatial objects (Maass et al., 2015). Aerobic fitness is also associated with an elevated executive function, which is mediated by an increase in the gray matter volume in the PFC (Weinstein et al., 2012). A 12-week moderate-intensity walking exercise can increase FC of the PCC/precuneus in individuals with MCI (Chirles et al., 2017). In summary, both cognitive training and aerobic exercise can improve cognitive function, while the intrinsic mechanism of why older adults can benefit from cognitive training and aerobic exercise remains to be determined (Owen et al., 2010; ten Brinke et al., 2015).

Located within the ventromedial portion of the temporal lobe, the human’s entorhinal cortex (EC) has long been suggested as one of the major structures significantly related to cognitive function (Coutureau and Di Scala, 2009). Previous studies found that the EC-hippocampal (HPC) circuit plays a vital role in the formation and recall of memory, preserving both spatial information and temporal information about past events (Marks et al., 2020). EC lesions disrupt a variety of functions, including attention, working memory and spatial cognition, some of which are not affected by hippocampal lesions (Oswald et al., 2001; Winters and Bussey, 2005). Such dissociations suggest that the EC may not be a pure relay station between the cortex and the hippocampus (Coutureau and Di Scala, 2009). A previous study investigating EC’s FC (termed as “EC-FC”) in patients with vascular MCI (VaMCI) revealed that VaMCI subjects showed a significantly decreased FC between the right EC and the right inferior frontal gyrus, right middle frontal gyrus, bilateral precentral gyri, right postcentral gyrus, and right superior parietal lobule. These results suggest that the EC-FC changes and their correlations with neuropsychological measures may aid in the early diagnosis of cognitive decline in the aging process (Zuo et al., 2018). EC, as a crucial cognitive function-related region, together with its associated large-scale human brain cognitive FC network, could play an important role in normative and pathologic aging and may serve as an important imaging marker for therapeutic effect on aging.

In this study, we explored the long-term effects of cognitive training and aerobic exercise on EC-FC by using seed-based FC analysis. We previously observed that the plasticity of the white mater was modified after training at the 12-month follow-up (Cao et al., 2016b). We hypothesized that prominent EC-FC changes could be found after cognitive training or aerobic exercise. In addition, cognitive training mainly improves memory, and aerobic exercise has a positive effect on attention, processing speed, and executive function (Smith et al., 2010; van Balkom et al., 2020), which might be attributed to the fact that these interventions enhance the corresponding cognition domain through different neural pathways. Therefore, we hypothesized that cognitive training and aerobic exercise modulate EC-FC through different neural circuits. Furthermore, our previous studies found a correlation between training-derived improvement in global cognition and increased FC between the central executive network after cognitive training (Cao et al., 2016a). The reduced FC between the left lateral occipital cortex and right superior temporal gyrus was associated with improved executive function after aerobic exercise (Hsu et al., 2018). Above all, we hypothesized that training-related alterations in EC-FC would be correlated with improvements in cognitive performance.

## Methods

### Participants

The present study was conducted using a subsample derived from our previous trial focusing on behavioral gains from cognitive training or aerobic exercise in healthy older adults, using the same methods of recruitment, eligibility criteria, randomization, intervention and outcome assessment. The original trial employed a prospective, block-randomized, assessor-blinded, controlled and parallel design (registration number: ChiCTR-TRC-13004788). 94 participants were recruited from 18 “Juwei” (a sub-district neighborhood administration) in the Jing’an district (central area) in Shanghai, China. Eligibility criteria were as follows: age 65–75 years; education ≥ 1 year; no difficulties with hearing, vision, or communication; no severe physical disease or psychotic disorder; and no obvious cognitive decline (a score on the Chinese version of the Mini-Mental State Examination ≥ 19). Exclusion criteria included the following: obvious cognitive decline such as AD; history or clinical evidence of neurological disease or psychiatric disorders such as brain cancer, major depressive disorder, or schizophrenia. This study was approved by the Human Research Ethics Committees of Shanghai Mental Health Center, China (approval number: 2013-40). Written informed consent was obtained from the participants after the study procedures had been explained to them.

### Interventions

The eligible participants were randomly assigned to three groups: the cognitive training group, aerobic exercise group and control group. Cognitive training comprised of memory exercises (including verbal memory, episodic memory, and face-name association memory), reasoning, problem-solving/strategy, and visuospatial ability (map reading). Each session lasted 60 min. Participants in the aerobic exercise group began with a 10-min brisk walk and gradually increased their walking time by 5 min each week until a total of 45 min is accomplished. Before and after each brisk walk, participants performed a five-minute stretch exercise to warm up and cool down. Participants wore a wrist heart rate monitor (Mio Alpha, Physical Enterprises Inc.) during brisk walking to monitor heart rate changes in real time. Both intervention groups received a total quorum of 24 sessions over 12 weeks and all three groups attended lectures on healthy living monthly during the first 3 months and every 6 weeks thereafter until the 12-month follow-up.

### Clinical Measurements

All measurements were conducted at baseline and 12 months after the intervention. Immediate memory (list learning and story memory), visuospatial/constructional (figure copy and line orientation), language (picture naming and semantic fluency), attention (digit span and coding), and delayed memory (list recall, list recognition, story recall and figure recall) index scores were obtained from the Repeatable Battery for the Assessment of Neuropsychological Status (RBANS; Form A and Form B), which has been verified in terms of its good reliability and validity in Chinese community-dwelling older adults (Cheng et al., 2011). According to the manual, four points should be added to the original score of semantic fluency of version B to achieve equivalence as version B is more difficult than version A (Randolf, 1998). The total scale index score of the RBANS represents global cognition. The visual reasoning test score reflected reasoning ability. The Stroop Color Word Test (SCWT) was used to measure cognitive inhibition and executive function, while the Color Trials Test (CTT) was used to measure processing speed, sequencing and mental flexibility, and visual-motor skills (Cao et al., 2016b).

### Magnetic Resonance Imaging (MRI) Acquisition

Magnetic Resonance Imaging data was collected on a Siemens 3.0-Tesla Magnetom Verio scanner (Siemens Medical, Erlangen, Germany) at Shanghai Mental Health Center. Images were obtained using a standard 12-channel head coil. High-resolution T1-weighted anatomical images (repetition time [TR] = 1900 ms, echo time [TE] = 3.43 ms, flip angle = 9°, 160 transverse slices, field of view (FOV) = 240 × 240 mm^2^, matrix size = 256 × 256, slice thickness = 1 mm, voxel size = 0.9 × 0.9 × 1.0 mm^3^) were acquired using a magnetization prepared rapid gradient-echo sequence. Resting-state functional MRI (rs-fMRI) data were acquired using a single-shot, gradient-recalled echo planar imaging sequence (TR = 2000 ms, TE = 25 ms, flip angle = 90°, 32 transverse slices, FOV = 240 × 240 mm^2^, matrix size = 64 × 64, slice thickness = 5 mm). For each subject, a total of 155 volumes were acquired, resulting in a total scan time of 310 s. The subjects were instructed to close their eyes and asked to refrain from thinking about anything and be awake. During the scan, foam padding was used to minimize head motion and earplugs were used to reduce scanner noise for each participant. After the scan, the technicians evaluated the quality of the structural images. If any abnormalities were found in the images, the participants would undergo rescanning. All the MRI data were collected at baseline and 12 months after the interventions.

### Rs-fMRI Pre-processing

Rs-fMRI data were pre-processed and analyzed using the Statistical Parametric Mapping software (SPM12)^1^ and the Data Processing Assistant for Resting-FMRI (DPARSF V 4.3^2^). For the rs-fMRI data, the first five functional images were discarded to reduce the fluctuation of MRI signals during the initial stages of scanning. Then, the slice-timing, head motion correction, normalization by DARTEL and spatial smoothing (with an isotropic Gaussian kernel of a full-width-at-half-maximum (FWHM) of 8 mm) were performed. Besides, the irrelevant signals including mean white matter signal, mean cerebrospinal fluid (CSF) signal, 24 head motion parameters, and the linear trend were regressed out by using a multiple linear regression model. To reduce physiological interference, a band-pass filter (0.01 – 0.1 Hz) was applied on all functional images. Moreover, subjects with head motion exceeding 3 mm in the translation or 3° in rotation along any direction were discarded.

### Seed-Based FC Analysis

The bilateral EC regions of interest (ROIs) were selected as seed regions according to a connectivity-based parcellation brain atlas (Fan et al., 2016), with the labels #115 and #116 in the brain atlas being located at the left and right EC, respectively. The blood oxygenation level-dependent rs-fMRI time series of the voxels within the bilateral EC ROIs was extracted and then averaged across the voxels, resulting in a EC seed time series. For each subject, an FC map was obtained by calculating the Pearson correlation between the seed’s time series and the other voxel’s time series within the brain. Moreover, a Fisher’s R-to-Z transformation was performed to enhance the normality of the resultant data.

### Statistical Analysis

The statistical software package SPSS 17.0 (IBM Corporation, Somer, NY, United States) was used to compare demographic and neuropsychological measures. One-way analysis of variance (ANOVA), the non-parametric Kruskal-Wallis test and the Chi-squared test were performed to compare baseline characteristics between participants among the three groups. The general linear model (GLM) repeated measure analysis was used to investigate the effect on cognitive performance after the interventions. The model included the main effect for the time and group and a time × group interaction term.

Cohen’s d, as a measure of effect sizes of the t-test for means, was calculated using G^∗^Power 3.1.9.3 (©Franz Faul, Edgar Erdfelder, Albert-Georg Lang, and Axel Buchner, 2006, 2009, Faul et al., 2007) and the confidence intervals of Cohen’s d were calculated by JASP Version 0.9 (JASP Team [2021]. JASP [Version 0.9] [computer software]). The net effect size (NES) was used to compare the cognitive measures at the 12-month post-test to baseline scores and control group scores. Bias-corrected NES of the intervention group was defined as: [(intervention mean at 12-month post-test - intervention mean at baseline) - (control mean at 12-month post-test - control mean at baseline)] ÷ pooled standard deviation at baseline, before applying a bias correction factor 1–3÷[4^∗^(nI + nc-2)-1] (Morris, 2007). Cohen defines *d*-values of 0.2, 0.5 and 0.8 as small, medium and large effects, respectively (Faul et al., 2007).

To evaluate the training effect on EC-FC, a voxel-wise 3 (between – subject factor: cognitive training, aerobic exercise and control groups) × 2 (within – subject factor: baseline and 12 months after the interventions) repeated analysis of covariance (ANCOVA) was performed by controlling the age, sex and years of education. The calculation of ANCOVA was limited on a union mask, which generated from the union set of the one-sample t-test results of EC-FC in each group. Then, between-groups 2 (cognitive training and control group; aerobic exercise and control group; cognitive training and aerobic exercise) × 2 (baseline and 12 months after the interventions) ANCOVA were performed in the regions with significant interaction effect of 3 × 2 ANCOVA as the *post hoc* test. These analyses were calculated by SPM 12, and *p* < 0.005 was considered statistically significant. Furthermore, the two-sample t-test was used to compare differences in EC-FC to explore the between-group differences, and paired t-test was carried out to clarify the effect of training on EC-FC for each group.

The regions with a significant interaction effect were extracted as the ROIs for the correlation analysis. Pearson correlations were conducted to evaluate the relationship between the EC-FC values in these ROIs and neuropsychological assessments at baseline after controlling for age, sex and years of education. To evaluate whether the changed EC-FC was related to the altered cognitive performance, the Pearson correlation analysis was also conducted between the changes of EC-FC in the ROIs with a significant interaction effect and the alteration of neuropsychological assessments. The neuropsychological assessments were chosen according to the NES results, the cognition outcomes which indicated a positive effect after the interventions when compared with the control group were included into the correlation analysis.

## Results

### Demographic and Neuropsychological Tests

**FIGURE 1:**
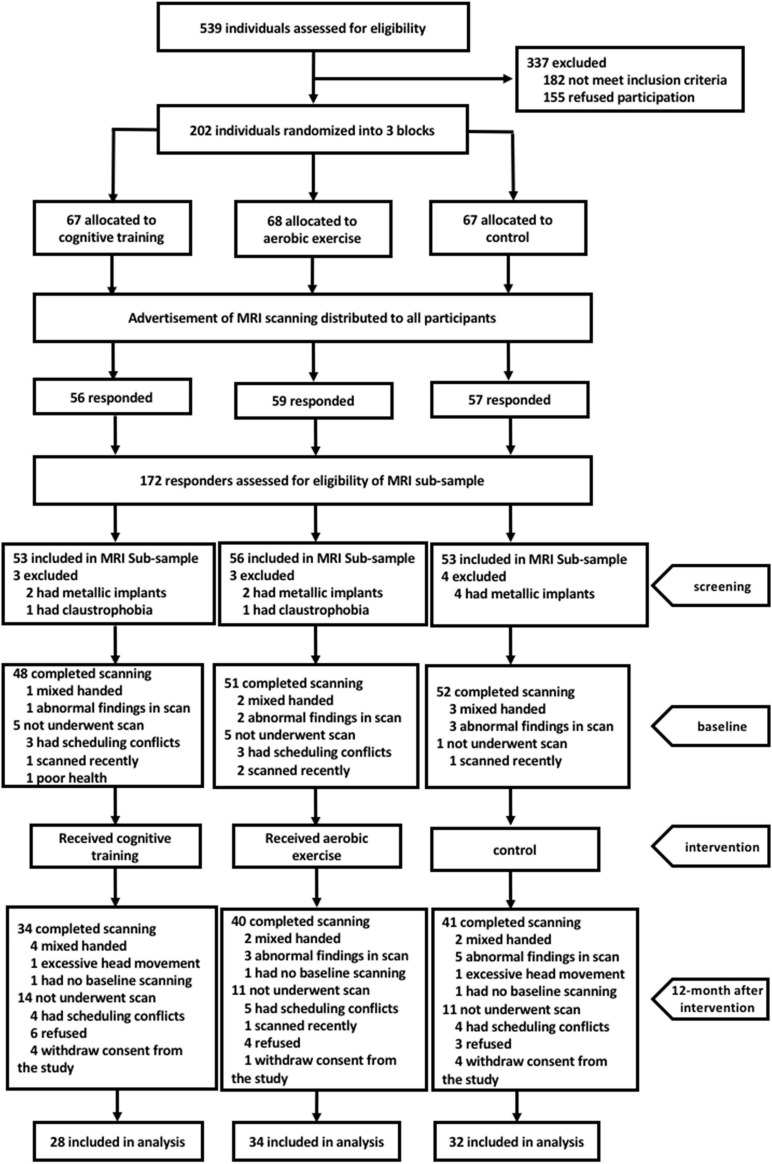
Flow chart showing the recruitment process of study participants.

A flow chart of the participant selection and inclusion procedures during the entire study is illustrated in Figure 1. Among the 539 eligible individuals who were contacted for participation from November 2013 to September 2014, 172 were included in the MRI subsample. Among them, 151 individuals subsequently completed the MRI scans; the remaining 21 were excluded due to the following: metal-related concerns, claustrophobia, history of recent scanning, conflicts of schedule, and poor health. Between baseline and a 12-month post-scan, 9 participants withdrew their consent from the study, 13 participants refused the scanning and the remaining 14 were excluded because of conflicts of schedule and history of recent scanning. Moreover, 21 participants were excluded from the statistical analyses because of abnormal MRI findings (*n* = 8), excessive head movements(*n* = 2), mixed handedness(*n* = 8), or lack of baseline scanning(*n* = 3). Overall, a total of 94 right-handed participants were used in our analysis (28 in the cognitive training group, 34 in the aerobic exercise group, and 32 in the control group). Demographic characteristics and neuropsychological scores are described in Table 1. There were no significant differences in the demographic data (age, sex, years of education) or any cognitive performance across the three groups at baseline.

**TABLE 1:** Demographic characteristics and cognitive measures of participants at baseline.

	Cognitive training group	Aerobic exercise group	Control group	F/χ^2^	*p* (2-tailed)
	(*n* = 28)	(*n* = 34)	(*n* = 32)		
Age, Mean ± SD (year)	68.20 ± 3.21	69.33 ± 2.42	68.41 ± 2.94	1.434	0.244
Male, *n* (%)	10 (35.7)	12 (35.3)	15 (46.9)	1.149	0.563
Education, years, Mean ± SD	11.48 ± 2.77	12.52 ± 2.88	11.94 ± 2.99	0.998	0.372
**Cognitive measures (Mean ± SD)**					
CMMSE (range 0–30)	27.61 ± 1.57	28.21 ± 1.51	28.03 ± 1.71	1.112	0.333
RBANS total score (range 0–160)	94.32 ± 11.61	94.74 ± 12.13	93.09 ± 12.79	0.159	0.854
Immediate memory (range 0–160)	87.04 ± 12.29	83.74 ± 16.16	82.22 ± 14.54	0.847	0.432
Visuospatial/Constructional (range 0–160)	100.32 ± 17.82	100.68 ± 16.38	101.44 ± 15.52	0.036	0.964
Language (range 0–160)^a^	97.39 ± 8.71	100.38 ± 8.42	95.84 ± 5.75	3.261	0.131
Attention (range 0–160)	99.07 ± 11.75	101.21 ± 14.57	100.50 ± 15.29	0.181	0.835
Delayed memory (range 0–160)	96.68 ± 12.65	95.47 ± 14.04	95.25 ± 13.88	0.094	0.910
SCWT Color interfere (second)	11.14 ± 6.80	16.21 ± 11.73	14.84 ± 10.13	2.089	0.130
SCWT Word interfere (second)	33.11 ± 16.40	43.61 ± 16.65	35.91 ± 22.80	2.573	0.082
Visual Reasoning Test (range 0–9)	7.25 ± 1.43	6.59 ± 1.71	7.06 ± 1.61	1.450	0.240
CTT-1 completion time (second)	91.39 ± 30.90	92.82 ± 33.78	85.03 ± 30.30	0.549	0.579
CTT-2 completion time (second)	150.39 ± 43.68	153.00 ± 54.54	133.03 ± 36.13	1.808	0.170

*CMMSE = Chinese version of Mini Mental State Examination; RBANS = Repeatable Battery for the Assessment of Neuropsychological Status (Form A); SCWT = Stroop Color Word Test; SCWT color interfere = Card C completion time-Card A completion time; SCWT word interfere = Card D completion time-Card B completion time. ^*a*^Kruskal-Wallis test.*

### Cognitive Performance at 1 Year After the Interventions

Table 2 shows the main effect of time, group and time × group interaction in terms of cognitive measures at the 12-month post-test. There were no significant time × group interactions in any cognitive performance across the three groups at 1 year after the interventions. Table 3 lists the effect size (Cohen’s *d*) of each group and the NES of the cognitive training and aerobic exercise groups on each cognitive measure at the 12-month post-test follow-up. The NES of cognitive training showed a small positive effect of training on the RBANS attention index score (NES = 0.343; 95% CI, 0.293∼0.387) and the RBANS delayed memory index score (NES = 0.237; 95% CI, 0.136∼0.295), while that of aerobic exercise had a small positive impact on the RBANS attention index score (NES = 0.316; 95% CI, 0.306∼0.330) and the RBANS visuospatial/constructional index score (NES = 0.239; 95% CI, 0.208∼0.271). The NES of cognitive training also showed a small to medium negative effect of training on the RBANS language index score (NES = −0.361; 95% CI, −0.374∼−0.365) and the SCWT color interfere time (NES = 0.562; 95% CI, 0.487∼−0.630), while that of aerobic exercise had a small to medium negative impact on the RBANS immediate memory index score (NES = −0.266; 95% CI, −0.322∼−0.214) and the SCWT color interfere time (NES = 0.336; 95% CI, 0.324∼−0.352).

**TABLE 2:** Cognitive performance at 1 year after the interventions.

Cognitive measures	Time	*p*	Group	*p*	Time × Group	*p*
RBANS total score	4.405	0.039	0.627	0.537	0.111	0.895
Immediate memory	91.867	<0.001	1.012	0.368	0.453	0.637
Visuospatial/Constructional	28.512	<0.001	0.063	0.939	0.044	0.957
Language	27.257	<0.001	0.696	0.501	2.019	0.139
Attention	16.370	<0.001	0.241	0.787	1.620	0.204
Delayed memory	59.623	<0.001	0.541	0.584	0.536	0.587
SCWT Color interfere	20.373	<0.001	1.492	0.230	1.788	0.173
SCWT Word interfere	0.319	0.574	2.518	0.122	1.112	0.333
Visual Reasoning Test	2.220	0.140	1.982	0.144	0.149	0.862
CTT-1 completion time	17.459	<0.001	0.917	0.403	0.191	0.827
CTT-2 completion time	20.644	<0.001	2.108	0.128	0.839	0.435

*RBANS = Repeatable Battery for the Assessment of Neuropsychological Status (Form B); SCWT = Stroop Color Word Test; SCWT color interfere = Card C completion time-Card A completion time; SCWT word interfere = Card D completion time-Card B completion time.*

**TABLE 3:** Net effect on cognitive performance at 1 year after the interventions.

Cognitive measures	Cognitive training group			Aerobic exercise group			Control group	
	*p*	Cohen’s d	Bias-corrected NES	*p*	Cohen’s d	Bias-corrected NES	*p*	Cohen’s d
		(95% confidential intervals)			(95% confidential intervals)			(95% confidential intervals)
RBANS total score	<0.001	0.702 (0.282, 1.111)	−0.049 (−0.070, −0.029)	<0.001	0.714 (0.326, 1.092)	−0.038 (−0.049, −0.027)	<0.001	0.752 (0.353, 1.140)
Immediate memory	<0.001	0.980 (0.521, 1.426)	−0.168 (−0.173, −0.165)	<0.001	0.881 (0.479, 1.274)	−0.266 (−0.322, −0.214)	<0.001	1.150 (0.696, 1.593)
Visuospatial/Constructional	0.011	−0.518 (−0.909, −0.119)	0.193 (0.187, 0.198)	0.009	−0.472 (−0.824, −0.114)	0.239 (0.208, 0.271)	<0.001	−0.714 (−1.098, −0.320)
Language	0.012	0.508 (0.110, 0.898)	−0.361 (−0.374, −0.345)	0.113	0.283 (−0.067, 0.629)	−0.584 (−0.656, −0.521)	<0.001	0.874 (0.460, 1.277)
Attention	0.008	0.539 (0.137, 0.931)	0.343 (0.293, 0.387)	0.005	0.511 (0.150, 0.865)	0.316 (0.306, 0.330)	0.288	0.191 (−0.160, 0.539)
Delayed memory	<0.001	0.973 (0.515, 1.418)	0.237 (0.136, 0.295)	<0.001	0.743 (0.358, 1.120)	0.010 (−0.019, 0.001)	<0.001	0.733 (0.337, 1.119)
SCWT Color interfere	<0.001	0.759 (0.332, 1.176)	0.562 (0.487, 0.630)	0.004	0.530 (0.167, 0.886)	0.336 (0.324, 0.352)	0.291	0.190 (−0.161, 0.538)
SCWT Word interfere	0.330	0.188 (−0.188, 0.560)	0.263 (0.233, 0.286)	0.089	−0.316 (−0.674, 0.048)	−0.235 (−0.247, 0.225)	0.663	−0.078 (−0.424, 0.270)
Visual Reasoning Test	0.310	0.195 (−0.180, 0.568)	0.089 (0.062, 0.114)	0.306	0.178 (−0.162, 0.516)	0.072 (0.065, 0.080)	0.557	0.105 (−0.243, 0.452)
CTT-1 completion time	0.040	−0.407 (−0.790, −0.018)	0.079 (0.059, 0.097)	0.016	−0.449 (−0.810, −0.082)	0.037 (0.034, 0.040)	0.010	−0.487 (−0.850, −0.116)
CTT-2 completion time	0.102	−0.320 (−0.697, 0.063)	0.180 (0.168, 0.191)	0.001	−0.616 (−0.991, −0.233)	−0.113 (−0.123, −0.103)	0.008	−0.502 (−0.867, −0.131)

*Cohen’s effect size calculated from t-test, positive value of RBANS, visual reasoning and negative value of SCWT, CTT for improvement at 12-month posttest; bias-corrected NES comparing the means and standard deviations of intervention groups and control group at 12-month posttest, positive value of RBANS, visual reasoning and negative value of SCWT, CTT is in favor of intervention group. RBANS = Repeatable Battery for the Assessment of Neuropsychological Status (Form B); SCWT = Stroop Color Word Test; SCWT color interfere = Card C completion time-Card A completion time; SCWT word interfere = Card D completion time-Card B completion time. For time-related indicators(such the completion time of SCWT and CTT), Cohen ’d<0 represents a better performance at 1-year follow-up when compared with baseline.*

### Interaction Effect in EC-FC

**FIGURE 2:**
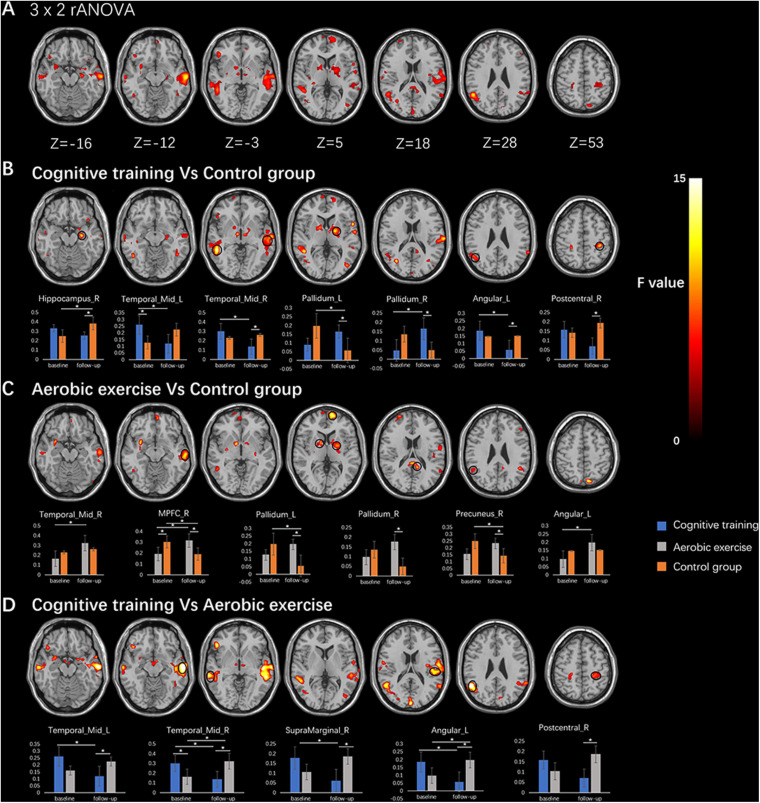
The results of rANCOVA in the entorhinal cortex functional connectivity (EC-FC). **(A)** The significant interaction effects of 3 × 2 rANCOVA were shown in the first line. **(B)** The significant interaction results of 2 × 2 rANCOVA among cognitive training and control group were shown in the second line, and the bar chart in the third line showed the results of the *post hoc* test within group/between-group. **(C)** The 2 × 2 rANCOVA among aerobic exercise and control group were shown in the fourth line, and the bar chart in the fifth line showed the results of the *post hoc* test within group/between-group. **(D)** The 2 × 2 rANCOVA among the cognitive training and aerobic exercise were shown in the sixth line, and the bar chart in the seventh line showed the results of the *post hoc* test within group/between-group. All interaction results showed *p* < 0.005, and * represented *p* < 0.05. The black circles showed the location of the ROIs.

The result of the interaction effect (group × time) in the mixed model analysis is shown in Figure 2. The 3 × 2 rANCOVA revealed significant interaction (*p* < 0.005) effects for EC-FC in some brain regions, mainly the right hippocampus, bilateral middle temporal gyrus, bilateral pallidum, right mPFC, right supramarginal gyrus, left angular gyrus and right postcentral gyrus. The *post hoc* test, 2 × 2 rANCOVA among the cognitive training and control group, showed significant interaction effects on the right hippocampus, bilateral middle temporal gyrus, bilateral pallidum, right supramarginal gyrus and left angular gyrus. In addition, the 2 × 2 rANCOVA among the aerobic exercise and control group showed significant interaction effects on the right hippocampus, right middle temporal gyrus, right mPFC, bilateral pallidum and right precuneus. We also performed the 2 (cognitive training and aerobic exercise) × 2 rANCOVA, and the significant interaction results were located on the bilateral middle temporal gyrus, left supramarginal gyrus, and left angular gyrus.

Furthermore, the cognitive training increased the connectivity between the EC and right pallidum, and decreased the connectivity between the EC and bilateral middle temporal gyrus, left supramarginal gyrus, and left angular gyrus. The connectivity between the EC and right middle temporal gyrus, right mPFC, and left angular gyrus were increased after aerobic exercise. In the control group, the increased connectivity between EC and right hippocampus was found after 12 months, with the decreased connectivity located on the right mPFC, left pallidum, and right precuneus gyrus. Compared to the control group, the cognitive training group showed a decreased EC-FC in the right hippocampus, right middle temporal gyrus, left angular gyrus, and right postcentral gyrus, and an increased EC-FC in the bilateral pallidum; the aerobic exercise group showed an increased EC-FC in the right mPFC, bilateral pallidum, and right precuneus. Interestingly, the cognitive training group showed a lower EC-FC in the bilateral middle temporal gyrus, right supramarginal, left angular gyrus, and right postcentral gyrus than the aerobic exercise group after training, and the cognitive training group did not show an increased EC-FC compared to the aerobic exercise group.

### Correlation Results

The RBANS visuospatial/constructional index score showed a positive correlation with EC-FC in the right mPFC at baseline (*r* = 0.24, *p* = 0.02, no correction), after controlling for sex and years of education.

**FIGURE 3:**
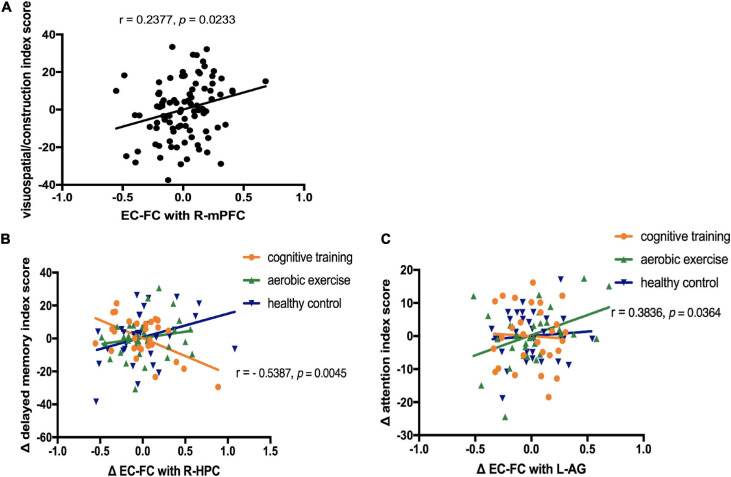
Correlation between the neuropsychological assessments and entorhinal cortex functional connectivity (EC-FC) values. **(A)** Linear correlation between the EC-FC values with the right medial prefrontal cortex(R-mPFC) and the visuospatial/constructional index score of the Repeatable Battery for the Assessment of Neuropsychological Status (RBANS) at baseline (*r* = 0.24, *p* = 0.02, no correction). **(B)** Linear correlation between the EC-FC value changes with the right hippocampus (R-HPC) and the change in the RBANS delayed memory index score in the cognitive training group [*r* = −0.54, *p* = 0.005, corrected for multiple comparisons (FDR, *q*<0.05)]. **(C)** Linear correlation between the EC-FC value changes with the left angular gyrus (L-AG) and the change in the RBANS attention index score in the aerobic exercise group (*r* = 0.38, *p* = 0.04, no correction).

We explored the relationships between the longitudinal EC-FC value changes and changes in the neuropsychological assessments (3 cognitive outcomes: the RBANS delayed memory index score, the RBANS attention index score, the RBANS visuospatial/constructional index score) by extracting the values of EC-FC rs-fMRI from the cluster showing significant time × group interaction (9 ROIs). As shown in Figure 3, in the cognitive training group, EC-FC value changes with the right hippocampus negatively correlated with the change in the RBANS delayed memory index score (*r* = −0.54, *p* = 0.005, corrected for multiple comparisons [FDR corrected, *q*<0.05]). In the aerobic exercise group, EC-FC value changes with the left angular gyrus positively correlated with the change in the RBANS attention index score (*r* = 0.38, *p* = 0.04, no correction).

## Discussion

A series of studies focusing on the positive effect of cognitive training or aerobic exercise have shown that the community-dwelling older adults can maintain cognitive improvement for at least 1 year after the interventions (Wolinsky et al., 2006; Lautenschlager et al., 2008). Cognitive training and aerobic exercise can induce plastic changes in the neural FC in healthy older adults, and these changes may underlie the positive effects of these two interventions (Chirles et al., 2017). Our study showed that compared to the cognitive training group, the aerobic exercise group had a significant increase in the EC-FC with the bilateral middle temporal gyrus/right supramarginal gyrus/left angular gyrus/right postcentral gyrus. Compared to the control group, the cognitive training group showed a decreased EC-FC in the right hippocampus/right middle temporal gyrus/left angular gyrus/right postcentral gyrus and an increased EC-FC in the bilateral pallidum, while the aerobic exercise group showed an increased EC-FC in the right mPFC/bilateral pallidum/right precuneus. The right mPFC showed a significant positive correlation between baseline EC-FC and RBANS visuospatial/constructional index score. In the cognitive training group, the right hippocampus showed a significant negative correlation between the EC-FC value changes and the change in the RBANS delayed memory index score. In the aerobic exercise group, EC-FC value changes with the left angular gyrus positively correlated with the change in the RBANS attention index score.

The abnormality in EC has been implicated in the early stage of AD (Khan et al., 2014). Both animal and human studies have demonstrated the indispensable role of EC in object memory and spatial memory (Chao et al., 2016; Shimada et al., 2017; Butler et al., 2019; Yeung et al., 2019). We found that the significant time × group interaction effect in EC-FC was detectable even at 12 months after the intervention, which implies that both cognitive training and aerobic exercise have long-term effects on neural plasticity changes in healthy older adults. Compared to the control group, the cognitive training group showed a decreased EC-FC in the right hippocampus/right middle temporal gyrus/left angular gyrus. EC cells may demonstrate a heterogeneous pattern of activity, which may be capable of driving the activity of HPC and thus contribute to memory encoding (Marks et al., 2020). The middle temporal gyrus contributes to the controlled retrieval of conceptual knowledge, while the angular gyrus is critical for the efficient automatic retrieval of specific semantic information (Davey et al., 2015). The expectation behind cognitive training is that it may improve cognitive capacity and thus increase neural efficiency by decreasing the demands on the neural system when completing the same task (Park and Bischof, 2013). The decreased EC-FC in our findings implies that cognitive training may improve neural efficiency and reduce resource utilization; therefore, the demands on the right hippocampus, right middle temporal gyrus, and left angular gyrus could be decreased for memory encoding and semantic retrieval. In addition, we found that the aerobic exercise group showed an increased EC-FC in the right mPFC and right precuneus when compared with those of the control group. These brain areas are associated with verbal memory and attention (Leon et al., 2010; Andrews-Hanna et al., 2014). These results indicate that aerobic exercise may have an influence on cognitive domains such as memory and attention mediated by the EC. The preceding evidence indicates that cognitive training and aerobic exercise affect brain structure and function as well as cognition via distinct mechanisms (Suo et al., 2016). In the present study, we found that aerobic exercise resulted in an increased rsFC between the EC and bilateral middle temporal gyrus, right supramarginal gyrus, left angular gyrus and right postcentral gyrus compared to the cognitive training group, which is partially consistent with the neuroplasticity changes in the bilateral middle temporal gyrus, right supramarginal gyrus, and right postcentral gyrus when following long-term aerobic exercise (Voss et al., 2013; Hsu et al., 2018; Tarumi et al., 2021). Overall, our results suggest that although both cognitive training and aerobic exercise can improve cognitive performance, the two may exert their neurological effects through different neural mechanisms.

Our results showed a significant positive correlation between baseline EC-FC with the right mPFC and the RBANS visuospatial/constructional index score among healthy older adults. RBANS visuospatial/constructional domain include the Rey-Osterrieth Complex Figure Test(copy) and the visual judgments of line orientation, which reflects visuospatial capacities (Winstanley et al., 2003; Schaadt et al., 2016). Previous studies have demonstrated that mPFC and EC are required for spatial cognition (Leon et al., 2010; Meister and Buffalo, 2018). Our results indicate that the EC-mPFC circuit may be involved in spatial cognition by mainly influencing visuospatial capacities among community-dwelling older adults. In the cognitive training group, we found that the right hippocampus showed a significant negative correlation between the EC-FC value changes and the change of RBANS delayed memory index score. In addition, EC-FC with the right hippocampus was decreased after 12 weeks of cognitive training when compared with the control group. The RBANS delayed memory index score included delayed recall of words, story and figure and the delayed recognition of words, which involved the assessment of episodic memory. Episodic memory tends to be sensitive to age, as older adults often show a reduced recall in most episodic memory tasks (Bherer, 2015). It has been reported that patients with MCI could benefit from cognitive training in episodic memory (Li et al., 2011). The encoding and retrieval of episodic memory is believed to be directly and indirectly related to the entorhinal-hippocampus(EC-HPC) circuit (Roy et al., 2017). Taken together, our results suggest that cognitive training can improve episodic memory in healthy older adults by decreasing the rsFC between the hippocampus and EC. This finding is consistent with a previous study showing that cognitive training could bolster neural efficiency in older adults (Iordan et al., 2020), which indicates that older adults may decrease the demand for the EC-HPC circuit after cognitive training as a result of the enhanced neural processing speed. We found that the EC-FC value changes in the left angular gyrus correlated positively with the change in the RBANS attention index score in the aerobic exercise group, and the EC-FC with the left angular gyrus was increased after 12 weeks of exercise. Our findings are consistent with those of previous studies where EC was associated with spatial attention (Wilming et al., 2018). Moreover, it has been reported that angular exhibits widespread patterns of FC with the temporal cortex, the default mode network, and other regions involved in perception, attention and spatial cognition (Andrews-Hanna et al., 2014). The results indicate that aerobic exercise may improve attention by strengthening the connection between the EC and the left angular gyrus.

In addition, we also observed several negative NES in behavioral performance, such as the RBANS language index score in the cognitive training group and the RBANS immediate memory index score in the aerobic exercise group. The results may be caused by the fluctuation of the cognitive level for the individuals at baseline and 1-year follow-up.

There are several limitations in the present study. First, it was limited by the small sample size. Based on the behavioral effect size of the present study, we calculated the required sample size by G-power. The results showed that the optimal sample size is 270, which could detect significant difference. Therefore, it may lack the statistical power to detect the time × group interactions in cognitive performance among the three groups. Second, although several correlation analyses could not reach the significant level after corrected for multiple comparisons, the correlations could reflect the tendency that the changed EC-FC was related to the altered cognitive performance. To further verify the results, large sample studies combined rs-MRI with other multimodal magnetic resonance are needed in the future. Moreover, although the training and social contacts of the two intervention groups were almost equal, using the active control or the social control group would be better for controlling the psychosocial factors of the group-based interventions (Miller et al., 2012). Besides, the absence of immediate MRI scans after the 3-month interventions were completed directly led to the lack of short-term training-related MRI changes, which might have helped us to further understand the underlying neural mechanism of the interventions. Future research should focus on further investigating the short-term and long-term effects of training on cognitive gain and neuroplasticity changes. Finally, fMRI tasks such as the N-back task could be performed in older adults for better detection of the functional activity of the brain regions during the cognitive performance.

In general, our results demonstrate that cognitive training and aerobic exercise significantly modulate the resting-state FC of EC through different pathways over a 12-month follow-up period. Older adults may gain benefits in episodic memory and attention from cognitive training and aerobic exercise, and the underlying mechanism involves the EC-FC with the right hippocampus and the left angular gyrus separately. Clarifying the neural regulation mechanism of cognitive training and aerobic exercise on EC-FC may help in developing new interventions to prevent age-related diseases, such as MCI and AD.

## Data Availability Statement

The raw data supporting the conclusions of this article will be made available by the authors, without undue reservation.

## Ethics Statement

The studies involving human participants were reviewed and approved by the Human Research Ethics Committees of Shanghai Mental Health Center, China. The patients/participants provided their written informed consent to participate in this study. Written informed consent was obtained from the individual(s) for the publication of any potentially identifiable images or data included in this article.

## Author Contributions

NG and HL analyzed the clinical and fMRI data and was responsible for writing the publication. CLi and XC designed the study and collected the data. TL and LJ collected the data. HZ and BZ analyzed the fMRI data and revised the manuscript. CLi and CLu are the guarantors of this work and took responsibility for the integrity of the data and the accuracy of the data analysis. All authors contributed to the article and approved the submitted version.

## Conflict of Interest

The authors declare that the research was conducted in the absence of any commercial or financial relationships that could be construed as a potential conflict of interest.

## Footnotes

1 http://www.fil.ion.ucl.ac.uk/spm

2 http://www.restfmri.net
