# Supplementary material for: Different Modulatory Effects of Cognitive Training and Aerobic Exercise on Resting State Functional Connectivity of Entorhinal Cortex in Community-Dwelling Older Adults
Source: Front Aging Neurosci. 2021 May 31;13:655245. doi: 10.3389/fnagi.2021.655245 (PMC8200543; doi:10.3389/fnagi.2021.655245)
Supplement: Supplementary file 1 [file Data_Sheet_1.pdf]

Supplementary

	Cognitive training group (n=55)				Aerobic exercise group (n=63)				Control group (n=62)			
	included in MRI analysis (n=28)	not included in MRI analysis (n=27)	t/χ^2	*p*(2-tailed)	included in MRI analysis (n=34)	not included in MRI analysis (n=29)	t/χ^2	*p*(2-tailed)	included in MRI analysis (n=32)	not included in MRI analysis (n=30)	t/χ^2	*p*(2-tailed)
Age, Mean±SD(year)	68.20±3.21	68.67±2.87	-0.569	0.572	69.33±2.42	68.41±2.84	1.394	0.168	68.41±2.94	69.20±2.99	-1.048	0.299
Male, n (%)	10(35.7)	11(40.7)	0.147	0.701	12(35.3)	11(37.9)	0.047	0.828	15(46.9)	16(53.3)	0.258	0.611
Education, years, Mean ± SD	11.48±2.77	12.41±2.79	-1.234	0.222	12.52±2.88	11.55±2.56	1.393	0.169	11.94±2.99	12.55±2.47	-0.875	0.385
RBANS total score	94.32±11.61	92.04±12.98	0.689	0.494	94.74±12.13	91.90±12.05	0.929	0.357	93.09±12.79	91.27±11.40	0.592	0.556

Table 1 Demographic characteristics and cognitive assessment for participants included and not included in MRI analysis at baseline

RBANS=Repeatable Battery for the Assessment of Neuropsychological Status (Form A)

	Cognitive training group (n=55)				Aerobic exercise group (n=63)				Control group (n=62)			
	included in MRI analysis (n=28)	randomly assigned (n=55)	t/χ^2	*p*(2-tailed)	included in MRI analysis (n=34)	randomly assigned (n=63)	t/χ^2	*p*(2-tailed)	included in MRI analysis (n=32)	randomly assigned (n=62)	t/χ^2	*p*(2-tailed)
Age, Mean±SD(year)	68.20±3.21	68.43±3.03	-0.320	0.750	69.33±2.42	68.91±2.64	0.779	0.438	68.41±2.94	68.79±2.97	-0.593	0.554
Male, n (%)	10(35.7)	21(38.2)	0.048	0.826	12(35.3)	23(36.5)	0.014	0.905	15(46.9)	31(50.0)	0.082	0.774
Education, years, Mean ± SD	11.48±2.77	11.94±2.79	-0.703	0.484	12.52±2.88	12.07±2.76	0.744	0.459	11.94±2.99	12.23±2.75	-0.480	0.632
RBANS total score	94.32±11.61	93.20±12.24	0.401	0.689	94.74±12.13	93.43±12.08	0.508	0.613	93.09±12.79	92.21±12.07	0.330	0.742

Table 2 Demographic characteristics and cognitive assessment for participants included in MRI analysis and randomly assigned at baseline

RBANS=Repeatable Battery for the Assessment of Neuropsychological Status (Form A)
